# The Census Bulletin

**Published:** 1895-04

**Authors:** 


### Editorial, Etc.

### THE CENSUS BULLETIN.

This JOURNAL had for ready reference in 1890, the correspondence between Dr. Thos. Brian Gunning and the Census Bureau of 1880, published in the Jan. number, 1881. An historical sketch of the opposition to the classification of dentists as manufacturers by the Census Office of 1890 appeared in the March number of the JOURNAL. In order to make the data as complete as possible, there is quoted below from the "extra census bulletin No. 67" what the tables show in part as to mechanical dentistry and dentists' materials, separately, and the explanation of the division of manufacturers for dis continuing its efforts to obtain reports.

1st. capital, excluding the value of hired property, under the subheads of land; buildings; machinery, tools, and implements, and live assets; 2d, miscellaneous expenses; 3d, average number of employes and total wages, the employes being subdivided into classes of officers, firm members and clerks, operatives, skilled and unskilled, and pieceworkers, the males, females, and children of each class being shown separately; 4th, cost of materials used; 5th, value of products.

A large number of reports were received from dentists, which purported to represent only the mechanical work, but it was evident that in many cases operative dentistry had been included. This fact, combined with the strenuous objection on the part of the profession to give any information whatever of the character required by the census schedule, on the ground that the census law did not seek to secure the returns for professional services, caused the office to discontinue its efforts to obtain further reports for the mechanical branch of the profession. The statistics published are those which were secured prior to the objection referred to.

### MECHANICAL DENTISTRY.

Number of establishments reporting, 3,214. Value of plant: Land, $453,523; Buildings, $438,849; machinery, tools and implements, $1,551,176. Live assets, $870,592. Miscellaneous expenses, $870,592. Cost of materials used, $1,475,255. Value of products, including receipts from custom work and repairing, $7,864,299.

AVERAGE NUMBER OF EMPLOYEES AND TOTAL WAGES.

Employees, 4,737. Wages, $3,481,189.

DENTISTS' MATERIALS.

Number of establishments reporting, 24. Value of plant: Land, $83,050; buildings, $389,050; machinery, tools and implements, $351,315. Miscellaneous expenses, $406,125. Cost of materials used, $993,855. Value of products, including receipts from custom work and repairing, $2,594,888.

AVERAGE NUMBER OF EMPLOYEES AND TOTAL WAGES.

Employees, 1,214. Wages, $867,626.

[Live assets includes raw materials; stock in process and finished products on hand, and cash, bills, and accounts receivable. Miscellaneous expenses includes rent for tenancy; taxes (including internal revenue); insurance; repairs, ordinary, of buildings and machinery; amount paid contractors; interest paid on cash used in the business, and all sundries not elsewhere reported. Cost of materials used includes fuel; rent of power and heat, and mill supplies.] R. G.

